# Hemodynamic Response, Coughing and Incidence of Cerebrospinal Fluid Leakage on Awakening with an Endotracheal Tube or Laryngeal Mask Airway in Place after Transsphenoidal Pituitary Surgery: A Randomized Clinical Trial

**DOI:** 10.3390/jcm10132874

**Published:** 2021-06-28

**Authors:** Paola Hurtado, Javier Tercero, Marta Garcia-Orellana, Joaquim Enseñat, Luis Reyes, Gemma Cabedo, Jose Rios, Enrique Carrero, Nicolas de Riva, Jaume Fontanals, Isabel Gracia, Isabel Belda, Ana M. Lopez, Neus Fabregas, Ricard Valero

**Affiliations:** 1 Department of Anesthesiology, Hospital Clínic de Barcelona, University of Barcelona, 08036 Barcelona, Spain; phurtado@clinic.cat (P.H.); jtercero@clinic.cat (J.T.); magarciao@clinic.cat (M.G.-O.); gcabedo@clinic.cat (G.C.); ecarrero@clinic.cat (E.C.); nderiva@clinic.cat (N.d.R.); jfonta@clinic.cat (J.F.); igracia@clinic.cat (I.G.); ibelda@clinic.cat (I.B.); analopez@clinic.cat (A.M.L.); fabregas@clinic.cat (N.F.); 2 Department of Neurosurgery, Hospital Clínic de Barcelona, University of Barcelona, 08036 Barcelona, Spain; jensenat@clinic.cat (J.E.); lareyes@clinic.cat (L.R.); 3 Biostatistics and Data Management Platform, Hospital Clínic de Barcelona, University of Barcelona, Barcelona,08036, Spain; Jose.Rios@uab.cat; 4 Institut d’Investigacions Biomèdiques August Pi i Sunyer (IDIBAPS), 08036 Barcelona, Spain; 5 Centro de Investigación Biomédica en Red de Salud Mental (CIBERSAM), 08036 Barcelona, Spain

**Keywords:** laryngeal mask airway, neuroanesthesia, awakening, cerebral hemodynamic response, systemic hemodynamic response, cerebrospinal fluid leakage

## Abstract

We aimed to compare systemic and cerebral hemodynamics and coughing during emergence after pituitary surgery after endotracheal tube (ETT) extubation or after replacing ETT with a laryngeal mask airway (LMA). Patients were randomized to awaken with an ETT in place or after replacing it with an LMA. We recorded mean arterial pressure (MAP), heart rate, middle cerebral artery (MCA) flow velocity, regional cerebral oxygen saturation (SrO_2_), cardiac index, plasma norepinephrine, need for vasoactive drugs, coughing during emergence, and postoperative cerebrospinal fluid (CSF) leakage. The primary endpoint was postoperative MAP; secondary endpoints were SrO_2_ and coughing incidence. Forty-five patients were included. MAP was lower during emergence than at baseline in both groups. There were no significant between-group differences in blood pressure, nor in the number of patients that required antihypertensive drugs during emergence (ETT: 8 patients (34.8%) vs. LMA: 3 patients (14.3%); *p* = 0.116). MCA flow velocity was higher in the ETT group (e.g., mean (95% CI) at 15 min, 103.2 (96.3–110.1) vs. 89.6 (82.6–96.5) cm·s^−1^; *p* = 0.003). SrO_2_, cardiac index, and norepinephrine levels were similar. Coughing was more frequent in the ETT group (81% vs. 15%; *p* < 0.001). CSF leakage occurred in three patients (13%) in the ETT group. Placing an LMA before removing an ETT during emergence after pituitary surgery favors a safer cerebral hemodynamic profile and reduces coughing. This strategy may lower the risk for CSF leakage.

## 1. Introduction

The transnasal transsphenoidal endoscopic approach to the sella turca for pituitary surgery and the expanded endonasal approach to the skull base have both improved over the last decade [1,2]. The goal of anesthetic management in pituitary surgery is to guarantee the adequate depth of anesthesia, control arterial blood pressure to enhance endoscopic visibility in the surgical field, and ensure a smooth awakening to prevent bleeding associated with Valsalva maneuvers or hypertension. Recovery from general anesthesia usually involves sympathetic activation and catecholamine release, which increase blood pressure and heart rate and, thus, elevate cerebral blood flow and oxygen consumption. There is some evidence that coughing and agitation during emergence and poor hemodynamic control during endotracheal extubation in neurosurgical patients undergoing brain surgery may lead to the development of brain complications as edema or hemorrhage [3]. Measures to prevent agitation, hypertension, shivering, and coughing are therefore well justified in neurosurgical patients [4,5].

Laryngeal mask airway (LMA) removal has been reported to have a better safety profile than tracheal extubation, with lower incidence of coughing, retching, and laryngospasm [6]. In this context, replacing an endotracheal tube (ETT) with an LMA at the end of a procedure has been recommended to allow for smooth emergence in at-risk extubations [7], in patients with highly irritable airway or in surgical procedures, such as after neurosurgery, where cardiovascular stimuli during extubation are to be avoided. However, few studies have been conducted to test the effect of this recommendation. Our previous results showed that neurosurgical patients undergoing brain surgery emerged from anesthesia with a more favorable hemodynamic profile, a lower incidence of coughing, and less cerebral hyperemia when an LMA replaced the ETT at the end of surgery but before emergence after undergoing a supratentorial craniotomy [8].

The transnasal transsphenoidal endoscopic approach has been reported to have less associated morbidity than open surgery [9,10], but postoperative cerebrospinal fluid (CSF) leakage remains a major limitation of this technique [11]. The incidence of CSF leakage was 8% in a patient series in our hospital [2]. Studies have linked this complication to postoperative coughing and lower airway disease [12].

The aim of this randomized controlled trial was to study the effect on the emergence of placing an LMA before removal of the ETT in the minimally invasive endoscopic endonasal transsphenoidal pituitary surgery. To this end, we compared systemic and cerebral hemodynamic variables and cough incidence during emergence from general anesthesia under two conditions: after ETT extubation, according to standard procedure, or after replacement of the ETT with an LMA just before extubation. A secondary objective was to assess the effect of this approach on the incidence of CSF leakage in these patients.

## 2. Materials and Methods

This prospective single-site randomized, open-label parallel trial was approved by the research ethics committee of Hospital Clínic de Barcelona (file number HCB/2016/0781) and registered at clinicaltrials.org (NCT02988804) on 9 December 2016. A data analysis and statistic plans were written and posted on ClinicalTrials.gov and in the institutional review board (research ethics committee of Hospital Clínic de Barcelona) files before data were accessed. The study was performed according to the Declaration of Helsinki Criteria, and this manuscript adheres to the CONSORT guidelines. Patients provided written informed consent.

Adult patients undergoing elective endoscopic endonasal transsphenoidal pituitary surgery were recruited from February 2017 until September 2019. We applied the following exclusion criteria before randomization: anticipated difficult airway (severe acromegaly, limited mouth opening) or unexpected Cormack–Lehane grade IV detected during laryngoscopy, risk of bronchial aspiration (e.g., gastroesophageal reflux disease or lower cranial nerve palsy), uncontrolled arterial hypertension during preoperative assessment. Surgical complications (e.g., severe bleeding, surgical approach modification) after randomization but before extubation were also exclusion criteria.

Patient characteristics (age, body mass index, sex) and relevant aspects of medical history were recorded. We also conducted a cough test during the pre-anesthetic evaluation [13]. It consisted of inviting the patient to take a deep breath and a unique coughing effort. Coughing more than once may reflect the onset of chronic pulmonary obstructive disease (COPD) exacerbation or upper-airway infection.

### 2.1. Anesthetic Procedure

In the operating room, patients were premedicated with intravenous midazolam (1–2 mg). Standard monitoring consisted of electrocardiography, pulse oximetry, continuous arterial pressures (S/5; Datex Ohmeda, Helsinki, Finland), depth of anesthesia (bispectral index) (BIS Brain Monitoring System; Covidien, Mansfield, MA, USA), neuromuscular blockade (response to train-of-four stimulation), temperature and urine output, cerebral regional oxygen saturation (SrO_2_) (INVOS 5100C Cerebral/Somatic Oximeter; Minneapolis, MN, USA), and cardiac index (LiDCOplus™; LiDCO, London, UK). A transcranial Doppler ultrasound monitor (Intraview; Rimed, Singen, Germany) was fixed at the temporal window to monitor middle cerebral artery (MCA) flow velocity during induction and emergence.

General anesthesia was provided with an intravenous site-effect, target-controlled perfusion of 4 µg mL^−1^ propofol, 2 ƞg mL^−1^ remifentanil (Orchestra Infusion Workstation, Primea Base; Fresenius Vial, Bad Homburg v.d.H., Germany), and an intravenous perfusion of rocuronium. Propofol and remifentanil infusions were set to maintain the BIS index between 40 and 60. Rocuronium doses were given to maintain a response between T0 and T1 in train-of-four stimulation. After intravenous injection of 1.5 mg kg^–1^ of lidocaine, we performed direct laryngoscopy and orotracheal intubation with a reinforced ETT (Lo-Contour Oral/Nasal cuffed tracheal tube; Mallinckrodt, Covidien, Tullamore, Ireland) in all patients. Tube sizes were assessed by the anesthetist according to a patient’s sex and weight. We recorded the Cormack–Lehane grade, the number of intubation attempts, and the need for additional equipment (tube introducer, videolaryngoscope). The ventilator (Primus; Dräger Medical Hispania, Madrid, Spain) parameters were set to maintain normocapnia and partial pressure of oxygen in arterial blood of 150–200 mm Hg. A pharyngeal tamponade was inserted to prevent the passage of blood to the stomach during surgery. One puff of xylometazoline 0.05% nasal spray was applied every 5 min for 1 h before surgery in the ward. All surgical procedures were performed by the same neurosurgeon (J.E.).

During the surgical procedure, arterial blood pressure was kept low, at about 20% below a patient’s baseline but always with a mean arterial pressure (MAP) above 50 mm Hg; the SrO_2_ was kept above 50 and no lower than 20% below baseline. Antihypertensive agent (urapidil) was administered when needed, and the doses were recorded. During postoperative period, we treated any hypertensive episode with urapidil.

At the end of surgery, with general anesthesia and muscle relaxation still in effect, we administered paracetamol (1 g) and ondansetron (4 mg) through the intravenous line and removed the pharyngeal tamponade.

### 2.2. Randomization

The first author (P.H.) opened a sequentially numbered, sealed envelope with patient allocation to either the standard procedure (tracheal extubation, ETT group) or replacement of the tracheal tube with a Proseal LMA mask (Laryngeal Mask Co. Ltd., Le Rocher, Victoria, Mahe, Seychelles) (LMA group) before emergence from anesthesia. We used a software-generated randomization list in a 1:1 ratio and unstratified blocks of four patients. The anesthetist was not blind to group assignment.

### 2.3. Study Procedures

In both groups, just after surgery ended, the pharyngeal tamponade was withdrawn, and careful aspiration of pharyngeal secretions was performed.

In the ETT group, just after surgery ended, intravenous infusions of propofol, remifentanil and rocuronium were stopped, and sugammadex (200 mg) was given to reverse the neuromuscular blockade (train-of-four responses were 1–2/4 at this time). Full recovery of train-of-four responses was documented. When the patient was breathing spontaneously and could follow simple commands, the tube was removed.

In the LMA group, after withdrawing the pharyngeal tamponade and aspirating pharyngeal secretions, with the patient still under general anesthesia, a LMA was inserted (size 4 or 5, according to the manufacturer’s recommendations), using a guided Bailey technique [14,15]. Specifically, the anesthetist first inserted a suction catheter 8–10 cm beyond the distal end of the drainage tube of the LMA to be used, and then inserted the mask behind the ETT, allowing the suction catheter to enter the esophagus and guiding the tip of the LMA into the correct position. After the cuff of the LMA was inflated to a pressure of 60 cm H_2_O with a cuff manometer (VBM Medizintechnik GmbH. Sulz, Germany), the ETT cuff was deflated and the tube was removed. Ventilation continued with the same parameters through the LMA. Intravenous infusions of propofol, remifentanil, and rocuronium were then stopped, and sugammadex (200 mg) was given, and full recovery of train-of-four responses was documented. Gentle manual ventilator assistance was provided until the patient resumed spontaneous breathing and responded to simple commands. The LMA was then removed. Ondansetron to prevent nausea was prescribed in the postoperative period.

Hemodynamic variables (blood pressure, cardiac index, heart rate, SrO_2_, MCA flow velocity) were recorded at 8 moments: baseline: before anesthetic induction; end of surgery: before extubation (ETT group) or before ETT replacement (LMA group); and throughout emergence at 1, 5, 10, 15, 30, and 60 min after extubation or LMA removal. The last blood pressure and heart rate measurements were taken in the postoperative recovery room. Respiratory variables (including end-tidal carbon dioxide concentration) and arterial blood gases were recorded during mechanical ventilation.

We measured norepinephrine plasma concentrations with a radioimmunoassay kit (Noradrenalin RIA, IBL, Hamburg, Germany) before induction and 30 min after extubation. The normal norepinephrine concentration range in our laboratory is 136–364 pg mL^−1^.

Any coughing episode during emergence was recorded. Postoperative epistaxis and CSF leakage, defined as clinically relevant rhinorrhea diagnosed by the neurosurgeon and requiring repair of the fissure in the nasal cavity, were recorded. The patients were expected to be discharged on the fourth postoperative day, and all were followed for 1 month after surgery.

### 2.4. Statistical Analysis

The primary endpoint was postoperative MAP. We calculated that we would need 21 patients in each group to detect between-group differences of 10 mm Hg in MAP, assuming a standard deviation (SD) of 11 mm Hg, with a type-1 error of 5% and power of 80%.

Mean (SD) results and 95% confidence intervals (CI) were calculated for each group. The global estimated group effects and 95% CI adjusted to baseline were calculated for each variable. Longitudinal models were constructed using the generalized estimating equation method to account for within-subject correlations over time by means of an unstructured correlation matrix. These models were used to analyze the effect of the intervention on MAP and the secondary endpoints (SrO_2_ and cough incidence) during emergence. The main independent factors were time from baseline (end of surgery) and study group (standard extubation vs. extubation after prior placement of an LMA). To evaluate the statistical significance of differences at each data recording time, the model, including the time interaction by group, was run again for each dependent variable. Bonferroni correction of *p* values was used to adjust for multiplicity in time-by-time analyses.

Homogeneity of groups at baseline was tested. A post hoc analysis was performed to compare the hypertensive patients to all the patients enrolled. All analyses were conducted with SPSS version 25 (IBM, Armonk, NY, USA), assuming the superiority of the intervention and a 2-tailed type I error of 5%.

## 3. Results

**Figure 1:**
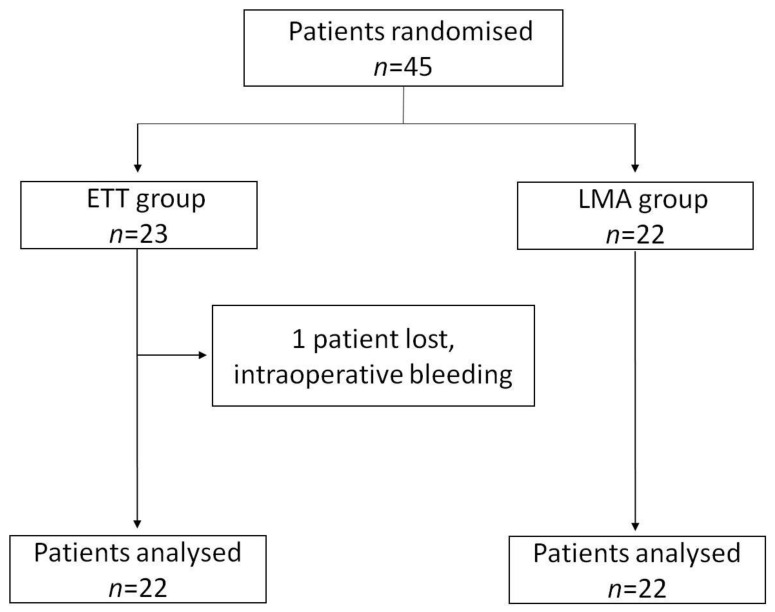
Flow diagram of patient enrolment, one loss, and data analysis.

Forty-five patients were randomized and included in the study. No differences in patient characteristics were found between groups at baseline (Table 1). The duration of surgery was 108 ± 35 min. The complexity of surgery was similar in both groups. One patient from the ETT group was lost to follow-up because extubation was delayed due to life-threatening intraoperative bleeding (carotid artery puncture) (Figure 1).

The Cormack–Lehane grade was II or lower in all cases, and all the ETTs were easily inserted. The LMA was successfully inserted on the first attempt in all LMA-group patients. No respiratory complications were observed during device replacement and emergence from anesthesia in the LMA group. Nor did partial pressure of oxygen in arterial blood or increases in end-tidal carbon dioxide concentration in expired air decrease during device replacement in the LMA group. No complications related to ETT replacement with an LMA were detected.

**Figure 2:**
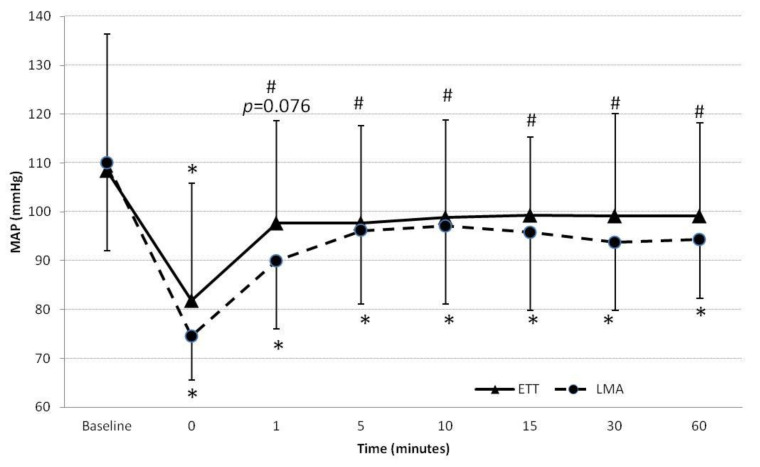
Changes in mean arterial blood pressure (MAP) in patients in the endotracheal tube (ETT) and laryngeal mask airway (LMA) groups. (* *p* < 0.001 with respect to baseline; ^#^ *p* < 0.05 with respect to baseline). Time 0 was the end of surgery, before extubation.

**Figure 3:**
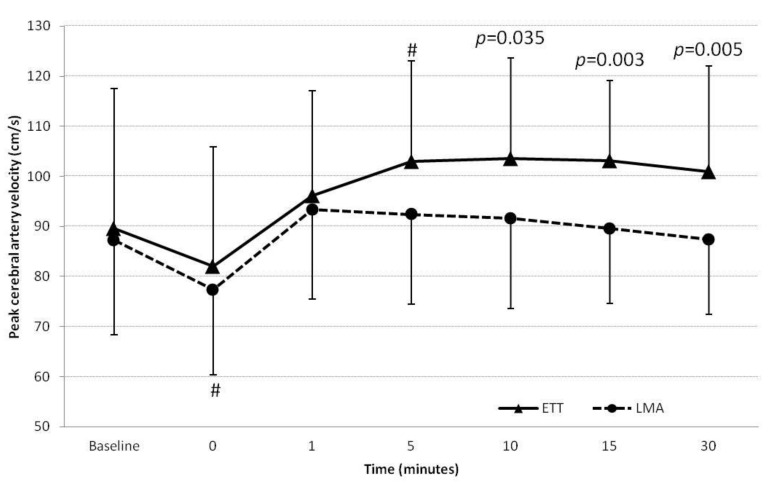
Changes in peak median cerebral artery (MCA) flow velocity (cm s^−1^) in patients in the endotracheal tube (ETT) and laryngeal mask airway (LMA) groups. (^#^ *p* < 0.05 with respect to baseline). Time 0 was the end of surgery, before extubation.

Hemodynamic variable changes are listed in Table 2 and plotted in Figure 2 and Figure 3. Thirteen patients (29.5%) required intraoperative antihypertensive drugs (urapidil dose ranging from 5 to 15 mg) to keep blood pressure low and within the range established for each patient. One patient needed ephedrine for an intraoperative hypotensive episode. Compared to baseline, blood pressure was significantly lower at all measured times during emergence from anesthesia in both groups. The greatest difference occurred at the end of surgery (*p* < 0.001) (Figure 2).

There were no significant between-group differences in blood pressure, nor in the number of patients that required antihypertensive drugs (urapidil dose ranging 10–20 mg) during emergence (ETT: eight patients (34.8%) vs. LMA: three patients (14.3%); *p* = 0.116) (Figure 2). Heart rate and the cardiac index did not significantly change from baseline and were similar in the two groups.

The peak MCA flow velocity measurements were significantly higher during ETT extubation compared to LMA removal at 5, 10, 15, and 30 min (Figure 3) (*p* = 0.003). Mean MCA flow velocity was also significantly higher at 15 min (*p* = 0.020), but SrO_2_ was similar in the two groups (Table 2).

Mean (SD) norepinephrine plasma levels were higher than at baseline at the end of emergence within each group, rising from 86.1 (65.9) to 103.7 (103.7) pg mL^−1^ in the ETT group and from 95.1 (59.2) to 175.5 (331.7) pg mL^−1^ in the LMA group. The difference between the groups was not significant (*p* = 0.329).

The analysis of hemodynamic variables in the subgroup of patients with a history of hypertension in each group showed that these patients did not differ from the full cohort of patients enrolled.

Significantly, more patients had coughing episodes in the ETT group than in the LMA group: 18 (81%) vs. 5 (15%) patients, respectively (*p* < 0.001). Postoperative epistaxis was recorded in four patients (18.2%) in the ETT group and three (15%) in the LMA group. Postoperative CSF leakage occurred in three patients (13%) in the ETT group; all of them underwent surgical repair and required an extended hospital stay of at least 1 week (range, 8–16 days). Despite no patients in the LMA group had this complication, the difference was not significant.

## 4. Discussion

Arterial blood pressure during emergence from anesthesia was stable in both groups but lower than baseline values, regardless of whether the ETT was replaced with an LMA or not before discontinuation of general anesthesia. Blood pressure tended to be nonsignificantly higher in the ETT group patients, even though they received some more antihypertensive treatment during emergence. However, the rise was not clinically relevant.

These findings contrast with the different hemodynamic profile of our previous study performed in patients after supratentorial craniotomy with a similar protocol [8]. In our former clinical trial on supratentorial craniotomy patients, baseline mean arterial pressure was lower than in the present study (about 20 mm Hg lower). We speculate that the preoperative administration of a quite high dose of xylometazoline could provoke a similar mild hypertensive effect in all included patients. Moreover, the present study population included 50% of patients with well-controlled chronic hypertension in both groups, compared with 18% in our previous study. On the other side, strict intraoperative control of arterial blood pressure, maintaining values within the lowest safe threshold to optimize the surgical field through the endoscopic procedure, is a fundamental clinical objective in this type of surgery. For all these reasons, the intraoperative tight control of blood pressure in this study could minimize the differences observed during emergence. Finally, our treatment of any hypertensive peak during emergence and prompt extubation after the resumption of spontaneous breathing may explain the absence of a difference in MAP between the two groups. Another possible explanation for the relatively similar low emergence of MAP values in both groups in the present study compared to our previous one is that pain immediately after transsphenoidal pituitary surgery may be less than after craniotomy, making blood pressure easier to control [16].

Although we did not find between-group differences in MAP during emergence, we found a higher mean MCA flow velocity in the ETT group. Since the anesthetic management and ventilation parameters were identical in both groups and no patient was hypercapnic at any moment during the study, the difference in MCA flow velocity was probably caused by slight differences in MAP, even though the group means did not differ significantly.

Measured norepinephrine levels were low and similar in both groups. Our patients were anesthetized with target-controlled intravenous propofol-remifentanil, so our observation of this stress hormone is consistent with a recent study that found that adrenocorticotropic hormone levels were significantly lower immediately after this type of anesthesia than after balanced anesthesia [17]. However, since a better stress hormone response has been reported with LMA use in laparoscopic gastric banding [18] and elective orthopedic surgery or general surgical procedures [19], we expected to see even lower values in the LMA group. We attribute the lack of difference in our study to the strict control of blood pressure and gentle extubation that is so necessary for neurosurgery.

Another main finding of our study was that patients in the LMA group had significantly fewer coughing episodes during emergence compared to the ETT group. This finding is in line with our previous study [8] and other studies that showed fewer episodes of postoperative cough, hypoxemia, and nausea or vomiting associated with LMA removal than with extubation [18,19]. Smooth emergence without coughing is particularly important in skull base surgery, justifying LMA placement before removing the ETT.

Reducing the risk of coughing must be balanced with the risk of aspiration and laryngospasm if some pharyngeal blood remnants from the surgical field reached the larynx. Cough can be reduced if patients have early extubation, with a deeper level of anesthesia, but in our case, this could have increased the risk of aspiration. In our study, patients were extubated, or the laryngeal mask was removed, when they had recovered a higher level of consciousness to minimize aspiration risk. In this way, laryngeal reflexes would have prevented patients from aspiration. Moreover, we removed the pharyngeal tamponade and performed a careful pharyngeal aspiration to remove any remaining blood and secretions. Finally, the insertion of the LMA in the right position before removing the orotracheal tube (Bailey maneuver), also helped minimize this potential complication.

Early extubation and LMA placement is not the only way to accomplish this goal. There are a number of other means to minimize the likelihood of coughing after any surgery, such as intravenous lidocaine, dexmedetomidine, that may also be considered [20].

The higher rate of coughing in the ETT group could be related to the three episodes of CSF leakage in this group. Hanba and colleagues [12] suggested that lower airway disease, commonly associated with frequent coughing, might be a risk factor for postoperative CSF rhinorrhea. The patients undergoing pituitary surgery who had this condition had almost a two-fold higher incidence of rhinorrhea compared to a cohort of disease-free patients. Their findings are consistent with those of a retrospective analysis of 2918 patients, 84 (2.9%) of whom were readmitted or reoperated for CSF leakage within 30 days of skull base surgery [21]. COPD was significantly associated with CSF leakage in those patients. In our study, the three patients with rhinorrhea required reintervention (flap closure of the fistula), prolonging the hospital stay. None of the three patients had COPD, but two of them were active smokers. Our study was not designed to find differences in CSF leakage The small sample size to demonstrate an association between coughing and this complication is a limitation of the study. Nevertheless, all our patients were operated on by the same team of surgeons, and the procedures were similar in both groups. Similar concerns may be considered for other types of surgeries where postoperative coughing may affect the outcome of the procedure, such as in facial plastic or aesthetic surgery, delicate sutures after visceral surgery, etc.

Replacing an ETT with an LMA at the end of the procedure has been recommended to allow smooth extubation in “at-risk” patients, such as smokers, asthmatics, and other patients with irritable airways or those in whom the surgical repair may be compromised by cardiovascular stimulation [7]. Emergence is the most intense moment for systemic and cerebral hemodynamic changes, even when extubation is performed meticulously [4]. Using the Bailey maneuver [14] in selected patients still under an adequate depth of anesthesia is a fast, effective and safe way to avoid apnea and a possible increase in PaCO_2_, which could disturb systemic and cerebral hemodynamics. However, it is not recommended in patients with anticipated or known difficult airway or at high risk of bronchial aspiration [7]. We used the Proseal LMA, a second generation supraglottic device, because it seals the airway better than first-generation devices, allows evacuation of gastroesophageal contents, and facilitates guided insertion. This LMA has been successfully used in other neurosurgical settings as lumbar spine microsurgery [22], ventriculoperitoneal shunt [23], and awake craniotomy [24].

Although the interest in reducing postoperative cough may also be important for other surgical procedures, our results in this very specific field may not be extrapolated.

## 5. Conclusions

In conclusion, placing an LMA before removing an ETT and ventilating through the mask during emergence from anesthesia after pituitary surgery favors a safer cerebral hemodynamic profile, although there was no difference in MAP nor in the need of antihypertensive drugs to keep MAP in the desired range. LMA also reduced coughing during an emergency. The possibility that this strategy may also lower the risk for CSF leakage warrants further investigation.

## Figures and Tables

**Table 1:** Patient characteristics.

	ETT Group	LMA Group
Age (year)	51.7 (14.9)	52.3 (11.4)
Sex (female/male)	12/11	13/9
BMI (kg m^−2^)	27.8 (3.6)	29.3 (5.5)
Cough test (positive)	None	None
Sleep apnea	2 (9%)	2 (9%)
Smokers	5 (21.7%)	2 (9.1%)
Acromegaly	3 (13.6%)	3 (13.6%)
Well-controlled hypertension	11 (50%)	11 (50%) ^1^

^1^ Values are expressed as mean (SD) or number of patients (percentage). ETT indicates endotracheal tube; LMA, laryngeal mask airway; BMI, body mass index.

**Table 2:** Hemodynamic data of patients in both groups.

			Emergence from Anesthesia					
	Baseline	Before Extubation	1 min	5 min	10 min	15 min	30 min	60 min
Systolic blood pressure (mm Hg)								
ETT	135.8 (28)	100.8 (24) *	123 (21) ^#^	123.9 (20)	127.4 (20)	126.4 (16)	124.7 (21) ^#^	126.3 (19)
LMA	138.6 (25)	91.9 (11) *	112.8 (23) *	120.1 (22) *	122.1 (23) *	120.4 (20) *	118.1 (18) *	118.7 (16) *
*p* value	0.719	0.101	0.120	0.550	0.413	0.273	0.255	0.146
Diastolic blood pressure (mm Hg)								
ETT	81.1 (16)	62.7 (17) *	72.3 (12) ^#^	71.4 (14) ^#^	70.3 (13) *	72.1 (13) *	73.6 (11) *	72.0 (11) *
LMA	81.6 (14)	57.1 (9) *	67.1 (12) *	72.1 (11) ^#^	72 (12) *	71.1 (14) ^#^	69.4 (11) *	69.9 (8) *
*p* value	0.924	0.165	0.146	0.854	0.667	0.784	0.182	0.423
Heart rate (beats per min)								
ETT	63.6 (11)	62.3 (15)	63.3 (17)	64 (16)	64 (14)	65.1 (13)	64 (11)	60.8 (11)
LMA	69.8 (12)	69.5 (12)	74.3 (13)	75.9 (12) ^#^	76.4 (11) ^#^	73.4 (11)	72.7 (11)	73 (11)
*p* value	0.065	0.067	0.011	0.004	0.001	0.023	0.008	0.000
Cardiac index (L min^−1^)								
ETT	2.9 (1)	2.1 (1) *	2.4 (1) ^#^	2.4 (1) ^#^	2.5 (1)	2.6 (1)	2.7 (1)	–
LMA	3.1 (1)	2.48 (1) ^#^	2.58 (1) ^#^	2.7 (1)	2.97 (1)	2.8 (1)	2.7 (1)	–
*p* value	0.543	0.184	0.723	0.477	0.240	0.580	0.990	–
SrO_2_, (%)								
ETT	67.7 (8)	66.8 (7)	69.1 (8)	69.3 (8)	70.0 (7)	70.9 (7)	70.5 (7)	–
LMA	65.5 (8)	65.8 (10)	68.9 (10)	70.9 (9) ^#^	70.1 (8)	70.4 (8)	70.4 (9)	–
*p* value	0.366	0.687	0.946	0.541	0.982	0.845	0.953	–
Mean MCA flow velocity (cm s^−1^)								
ETT	57.0 (15)	50.2 (17)	59.4 (17)	63.3 (20)	65.8 (24)	66.5 (18)	62.0 (18)	–
LMA	55.8 (13)	42.2 (12) *	57.7 (19)	56.6 (17)	58 (18)	55.7 (13)	55.2 (13)	–
*p* value	0.783	0.063	0.759	0.216	0.204	0.020	0.146	–

Values are expressed as mean (SD). ETT indicates endotracheal tube; LMA, laryngeal mask airway; MCA, middle cerebral artery; SrO_2_, regional cerebral oxygen saturation. *p* value: comparisons between groups; comparisons of group means for intraindividual differences with respect to baseline: * *p* < 0.001; ^#^  *p* < 0.05.

## Data Availability

The datasets used and analyzed during this study are available from the corresponding author on reasonable request.
